# On the origin and continuing evolution of SARS-CoV-2

**DOI:** 10.1093/nsr/nwaa036

**Published:** 2020-03-03

**Authors:** Xiaolu Tang, Changcheng Wu, Xiang Li, Yuhe Song, Xinmin Yao, Xinkai Wu, Yuange Duan, Hong Zhang, Yirong Wang, Zhaohui Qian, Jie Cui, Jian Lu

**Affiliations:** State Key Laboratory of Protein and Plant Gene Research, Center for Bioinformatics, School of Life Sciences, Peking University, Beijing 100871, China; State Key Laboratory of Protein and Plant Gene Research, Center for Bioinformatics, School of Life Sciences, Peking University, Beijing 100871, China; CAS Key Laboratory of Molecular Virology & Immunology, Institut Pasteur of Shanghai, Chinese Academy of Sciences, Shanghai 200031, China; Center for Biosafety Mega-Science, Chinese Academy of Sciences, Wuhan 430071, China; University of Chinese Academy of Sciences, Beijing 100049, China; CAS Key Laboratory of Molecular Virology & Immunology, Institut Pasteur of Shanghai, Chinese Academy of Sciences, Shanghai 200031, China; School of Life Sciences, Shanghai University, Shanghai 200444, China; State Key Laboratory of Protein and Plant Gene Research, Center for Bioinformatics, School of Life Sciences, Peking University, Beijing 100871, China; State Key Laboratory of Protein and Plant Gene Research, Center for Bioinformatics, School of Life Sciences, Peking University, Beijing 100871, China; State Key Laboratory of Protein and Plant Gene Research, Center for Bioinformatics, School of Life Sciences, Peking University, Beijing 100871, China; State Key Laboratory of Protein and Plant Gene Research, Center for Bioinformatics, School of Life Sciences, Peking University, Beijing 100871, China; State Key Laboratory of Protein and Plant Gene Research, Center for Bioinformatics, School of Life Sciences, Peking University, Beijing 100871, China; NHC Key Laboratory of Systems Biology of Pathogens, Institute of Pathogen Biology, Chinese Academy of Medical Sciences and Peking Union Medical College, Beijing 100730, China; CAS Key Laboratory of Molecular Virology & Immunology, Institut Pasteur of Shanghai, Chinese Academy of Sciences, Shanghai 200031, China; Center for Biosafety Mega-Science, Chinese Academy of Sciences, Wuhan 430071, China; State Key Laboratory of Protein and Plant Gene Research, Center for Bioinformatics, School of Life Sciences, Peking University, Beijing 100871, China

**Keywords:** SARS-CoV-2, virus, molecular evolution, population genetics

## Abstract

The SARS-CoV-2 epidemic started in late December 2019 in Wuhan, China, and has since impacted a large portion of China and raised major global concern. Herein, we investigated the extent of molecular divergence between SARS-CoV-2 and other related coronaviruses. Although we found only 4% variability in genomic nucleotides between SARS-CoV-2 and a bat SARS-related coronavirus (SARSr-CoV; RaTG13), the difference at neutral sites was 17%, suggesting the divergence between the two viruses is much larger than previously estimated. Our results suggest that the development of new variations in functional sites in the receptor-binding domain (RBD) of the spike seen in SARS-CoV-2 and viruses from pangolin SARSr-CoVs are likely caused by natural selection besides recombination. Population genetic analyses of 103 SARS-CoV-2 genomes indicated that these viruses had two major lineages (designated L and S), that are well defined by two different SNPs that show nearly complete linkage across the viral strains sequenced to date. We found that L lineage was more prevalent than the S lineage within the limited patient samples we examined. The implication of these evolutionary changes on disease etiology remains unclear. These findings strongly underscores the urgent need for further comprehensive studies that combine viral genomic data, with epidemiological studies of coronavirus disease 2019 (COVID-19).

## INTRODUCTION

SARS-CoV-2 was detected in late December 2019 in Wuhan, the capital of Central China's Hubei Province. Since then, it has rapidly spread across China and in other countries, raising major global concerns. This novel coronavirus, SARS-CoV-2, was named for the similarity of its structure to severe acute respiratory syndrome related coronaviruses. As of February 28, 2020, 78,959 cases of SARS-CoV-2 infection have been confirmed in China, with 2,791 deaths. Worryingly, there have also been more than 3,664 confirmed cases outside of China in 46 countries and areas (https://www.who.int/emergencies/diseases/novel-coronavirus-2019/situation-reports/), raising significant issues for successful containment. Further, the genomic sequences of SARS-CoV-2 viruses isolated from a number of patients share sequence identity higher than 99.9%, suggesting a very recent host shift into humans [1–3].

Coronaviruses are naturally hosted and evolutionarily shaped by bats [4,5]. Indeed, it has been postulated that most of the coronaviruses in humans are derived from the bat reservoir [6,7]. Several teams have recently confirmed the genetic similarity between SARS-CoV-2 and a bat betacoronavirus of the sub-genus *Sarbecovirus* [8–13]. The whole-genome sequence of the novel virus has 96.2% similarity to that of a bat SARS-related coronavirus (SARSr-CoV; RaTG13) collected in Yunnan province, China [2,14], but has low similarity to that of SARS-CoV (about 79%) or MERS-CoV (about 50%) [1,15]. It has also been confirmed that the SARS-CoV-2 uses the same receptor, the angiotensin converting enzyme II (ACE2), as the SARS-CoV [2]. Although the specific route of transmission from natural reservoirs to humans remains unclear [5,13], several studies have shown that pangolins may have provided a partial *spike* gene to SARS-CoV-2; the critical functional sites in the spike protein of SARS-CoV-2 are nearly identical to those identified in a virus isolated from a pangolin [16–18].

Despite these recent discoveries, several fundamental issues related to the evolutionary patterns and driving forces behind this outbreak of SARS-CoV-2 remain to be fully characterized [19–21]. Herein, we investigated the extent of molecular divergence between SARS-CoV-2 and other related coronaviruses and carried out population genetic analyses of 103 sequenced genomes of SARS-CoV-2. This work provides new insights into evolution of SARS-CoV-2 and its pattern of spread through the human population.

## RESULTS

### Molecular phylogeny and divergence between SARS-CoV-2 and related coronaviruses

For each annotated ORF in the reference genome of SARS-CoV-2 (NC_045512), we extracted the orthologous sequences in human SARS-CoV, four bat SARS-related coronaviruses (SARSr-CoV: RaTG13, ZXC21, ZC45, and BM48-31), one pangolin SARSr-CoV from Guangdong (GD), and six pangolin SARSr-CoV genomes from Guangxi (GX) [18] (Table S1). We aligned the coding sequences (CDSs) based on the protein alignments (see Materials and Methods). Most ORFs annotated from SARS-CoV-2 were found to be conserved in other viruses, except for *ORF8* and *ORF10* (Table 1). The protein sequence of SARS-CoV-2 *ORF8* shared very low similarity with those sequences in SARS-CoV and BM48-31, and *ORF10* had a premature stop codon in both SARS-CoV and BM48-31 (Fig. S1). A one-base deletion caused a frame-shift mutation in *ORF10* of ZXC21 (Fig. S1).

**Table 1.** The molecular divergence between SARS-CoV-2 and related viruses.

Gene	Aligned Length (nt)	RaTG13	GD Pangolin-CoV	GX Pangolin-CoV	SARSr-CoV ZC45	SARS-CoV	SARSr-CoV BM48–31
Genomic Average	28734	0.008/0.17 (0.044)	0.025/0.469 (0.053)	0.055/0.722 (0.076)	0.044/0.549 (0.081)	0.113/0.926 (0.122)	0.143/1.15 (0.124)
*ORF10*	114	0.011/0 (NA)	0.011/0 (NA)	0.072/0.044 (1.637)	0.011/0 (NA)	-	-
*ORF3a*	825	0.009/0.157 (0.06)	0.025/0.287 (0.086)	0.066/0.518 (0.128)	0.052/0.508 (0.102)	0.188/0.918 (0.205)	0.271/0.923 (0.294)
*ORF6*	183	0/0.098 (0)	0.014/0.217 (0.063)	0.038/0.491 (0.077)	0.027/0.173 (0.158)	0.191/0.913 (0.209)	0.393/1.512 (0.26)
*ORF7a*	363	0.011/0.177 (0.061)	0.018/0.275 (0.066)	0.073/0.477 (0.153)	0.066/0.351 (0.188)	0.088/0.697 (0.126)	0.337/1.14 (0.296)
*ORF7b*	129	0.01/0 (NA)	0.02/0.455 (0.043)	0.17/0.436 (0.39)	0.029/0.181 (0.162)	0.155/0.401 (0.387)	0.264/NA (NA)
*ORF8*	363	0.021/0.07 (0.303)	0.032/0.303 (0.105)	0.099/1.015 (0.098)	0.03/0.603 (0.05)	-	-
*E*	225	0/0.018 (0)	0/0.037 (0)	0.006/0.096 (0.063)	0/0.056 (0)	0.027/0.166 (0.164)	0.043/0.352 (0.121)
*M*	666	0.004/0.186 (0.021)	0.014/0.298 (0.046)	0.025/0.372 (0.067)	0.016/0.283 (0.055)	0.07/0.576 (0.121)	0.109/1.292 (0.085)
*N*	1257	0.005/0.131 (0.039)	0.012/0.149 (0.08)	0.04/0.304 (0.132)	0.036/0.333 (0.108)	0.059/0.381 (0.155)	0.102/1.197 (0.085)
*orf1a*	13215	0.009/0.167 (0.054)	0.024/0.475 (0.052)	0.073/0.811 (0.09)	0.026/0.405 (0.063)	0.148/1.141 (0.129)	0.174/1.199 (0.145)
*orf1ab*	21288	0.007/0.152 (0.044)	0.018/0.487 (0.037)	0.055/0.776 (0.071)	0.031/0.527 (0.058)	0.105/0.962 (0.109)	0.125/1.108 (0.113)
*S* (*spike*)	3819	0.014/0.321 (0.043)	0.076/0.7 (0.11)	0.06/0.86 (0.07)	0.138/1.063 (0.13)	0.172/1.265 (0.136)	0.217/1.518 (0.143)

For each gene, the dN and dS values between SARS-CoV-2 and another virus are given, and the dN/dS (ω) ratio is given in the parenthesis.

**Figure 1.**
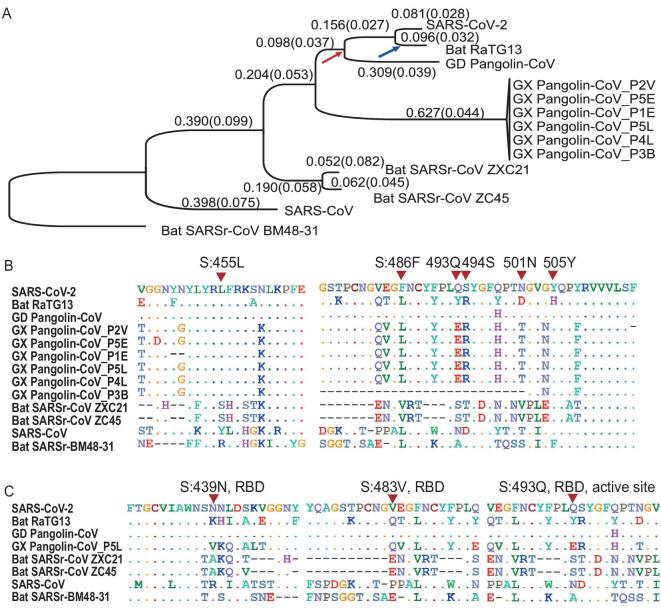
Molecular divergence and selective pressures during the evolution of SARS-CoV-2 and related viruses. (A) The phylogenetic tree of SARS-CoV-2 and the related Coronaviruses. The branch length (dS) is presented, and the dN/dS (ω) value is given in the parenthesis. The phylogenetic tree was reconstructed with the synonymous sites in the concatenated CDSs of nine conserved ORFs (*orf1ab, E, M, N, S, ORF3a, ORF6, ORF7a* and *ORF7b*). (B) Conservation of 6 critical amino acid residues in the spike (S) protein. The critical active sites are Y442, L472, N479, D480, T487, and Y491 in SARS-CoV, and they correspond to L455, F486, Q493, S494, N501, and Y505 in SARS-CoV-2 (marked with inverted triangles), respectively. (C) Three candidate positively selected sites (marked with inverted triangles) in the receptor-binding domain (RBD) of spike protein (S:439 N, S:483 V and S:493Q) and the surrounding 10 amino acids.

To investigate the phylogenetic relationship between these viruses at the genomic scale, we concatenated coding regions (CDSs) of the nine conserved ORFs (*orf1ab, E, M, N, S, ORF3a, ORF6, ORF7a,* and *ORF7b*) and reconstructed the phylogenetic tree using the synonymous sites (Fig. 1A). We also used CODEML in the PAML [22] to infer the ancestral sequence of each node and calculated the dN (nonsynonymous substitutions per nonsynonymous site), dS (synonymous substitutions per synonymous site), and dN/dS (ω) values for each branch (Fig. 1A). In parallel, we also calculated the pairwise dN, dS, and ω values between SARS-CoV-2 and another virus (Table 1).

The genome-wide phylogenetic tree indicated that SARS-CoV-2 was closest to RaTG13, followed by GD Pangolin SARSr-CoV, then by GX Pangolin SARSr-CoVs, then by ZC45 and ZXC21, then by human SARS-CoV, and finally by BM48-31(Fig. 1A). Notably, we found that the nucleotide divergence at synonymous sites between SARS-CoV-2 and other viruses was much higher than previously anticipated. For example, although the overall genomic nucleotides differ ∼4% between SARS-CoV-2 and RaTG13, the genomic average dS was 0.17, which means the divergence at the neutral sites is 17% between these two viruses (Table 1). Note that nonsynonymous sites are usually under stronger negative selection than synonymous sites, and calculating sequence differences without separating these two classes of sites may underestimate the extent of molecular divergence by several folds.

We found that the dS value varied considerably across genes in SARS-CoV-2 and the other viruses analyzed. In particular, the *spike* gene (*S*) consistently exhibited larger dS values than other genes (Table 1). This pattern became clear when we calculated the dS value for each branch in Fig. 1A for the *spike* gene versus the concatenated sequences of the remaining genes (Fig. S2). In each branch, the dS of *spike* was 2.29 ± 1.45 (mean ± SD) times as large as that of the other genes. This extremely elevated dS value of *spike* could be caused either by a high mutation rate or by natural selection that favors synonymous substitutions. Synonymous substitutions may serve as another layer of genetic regulation, guiding the efficiency of mRNA translation by changing codon usage [23]. If positive selection is the driving force for the higher synonymous substation rate seen in *spike*, we expect the frequency of optimal codons (FOP) of *spike* to be different from that of other genes. However, our codon usage bias analysis (Table S2) suggests the FOP of *spike* was only slightly higher than that of the genomic average (0.717 versus 0.698, see Materials and Methods). Thus, we believe that the elevated synonymous substitution rate measured in *spike* is more likely caused by higher mutational rates; however, the underlying molecular mechanism remains unclear.

Both SARS-CoV and SARS-CoV-2 bind to ACE2 through the RBD of the spike protein in order to initiate membrane fusion and enter human cells [1,2,24–28]. Five out of the six critical amino acid (AA) residues in RBD were different between SARS-CoV-2 and SARS-CoV (Fig. 1B), and a 3D structural analysis indicated that the spike of SARS-CoV-2 had a higher binding affinity to ACE2 than SARS-CoV [25]. Intriguingly, these same six critical AAs are identical between GD Pangolin-CoV and SARS-CoV-2 [16]. In contrast, although the genomes of SARS-CoV-2 and RaTG13 are more similar overall, only one out of the six functional sites are identical between the two viruses (Fig. 1B). It has been proposed that the SARS-CoV-2 RBD region of the spike protein might have resulted from recent recombination events in pangolins [16–18]. Although several ancient recombination events have been described in *spike* [29,30], it also seems likely that the identical functional sites in SARS-CoV-2 and GD Pangolin-CoV may actually result from coincidental convergent evolution [18].

If the functional AA residues in the SARS-CoV-2 RBD region were acquired from GD Pangolin-CoV in a very recent recombination event, we would expect the nucleotide sequences of this region to be nearly identical between the two viruses. However, for the CDS sequences that span five critical AA sites in the SARS-CoV-2 spike (ranging from codon 484 to 507, covering five adjacent functional sites: F486, Q493, S494, N501, and Y505; Fig. S3), we estimated dS = 0.411, dN = 0.019, and ω = 0.046 between SARS-CoV-2 and GD Pangolin-CoV. By assuming the synonymous substitution rate (*u*) of 1.67–4.67 × 10^−3^/site/year, as estimated in SARS-CoV [31], the recombination/introgression, if it occurred at all, would be estimated to happen approximately 19.2–53.7 years ago. Here, the formula $t\ = \ dS/( {u \times \ 2\ \times \ 2.29} )$ was used to calculate divergence time; note that the increased mutational rate of *spike* was considered for this calculation. Thus, it seems very unlikely that SARS-CoV-2 originated from the GD Pangolin-CoV due to a very recent recombination event. Rather, it seems more likely that a high mutation rate in *spike*, coupled with strong natural selection, has shaped the identical functional AA residues between these two viruses, as proposed previously [18]. Although these sites are maintained in SARS-CoV-2 and GD Pangolin-CoV, mutations may have changed the residues in the RaTG13 lineage after it diverged from SARS-CoV-2 (the blue arrow in Fig. 1A). In summary, the shared identity of critical AA sites between SARS-CoV-2 and GD Pangolin-CoV may be due to random mutations coupled with natural selection, rather than recombination.

### Selective constraints and positive selection during the evolution of SARS-CoV-2 and related coronaviruses

The genome-wide ω value between SARS-CoV-2 and other viruses ranged from 0.044 to 0.124 (Table 1), indicative of strong negative selection on the nonsynonymous sites. In other words, 87.6% to 95.6% of the nonsynonymous mutations were removed by negative selection during viral evolution. To determine the extent of positive selection, we concatenated the CDS sequences of 9 conserved ORFs in all the viruses in Fig. 1A and fitted the M7 (beta: neutral and negative selection) and M8 (beta + ω > 1: neutral, negative selection, and positive selection) model using CODEML (Materials and Methods). The M8 model (*lnL* = −104,813.732, *np* = 18) was a significantly better fit than the M7 (*lnL* = −105,063.284, *np* = 16) model (*P* < 10^−10^), suggesting that some AA substitutions were favored by positive Darwinian selection (but not necessarily in the SARS-CoV-2 lineage). Under the M8 model, 98.48% (*p_0_*) of the nonsynonymous substitutions were estimated under neutral evolution or purifying selection (0 ≤ ω ≤1), and 1.52% (*p_1_*) of the nonsynonymous substitutions were under positive selection (ω = 1.50). A Bayes Empirical Bayes (BEB) analysis suggested that 10 AA sites showed strong signals of positive selection, and, interestingly, three of these were located in the RBD of spike, including at one critical site (Fig. 1C and Fig. S4). Thus, although these coronaviruses were generally under very strong negative selection, positive selection was also responsible for the evolution of protein sequences. These putatively positively-selected sites deserve further functional studies.

### Mutations in 103 SARS-CoV-2 genomes

**Figure 2.**
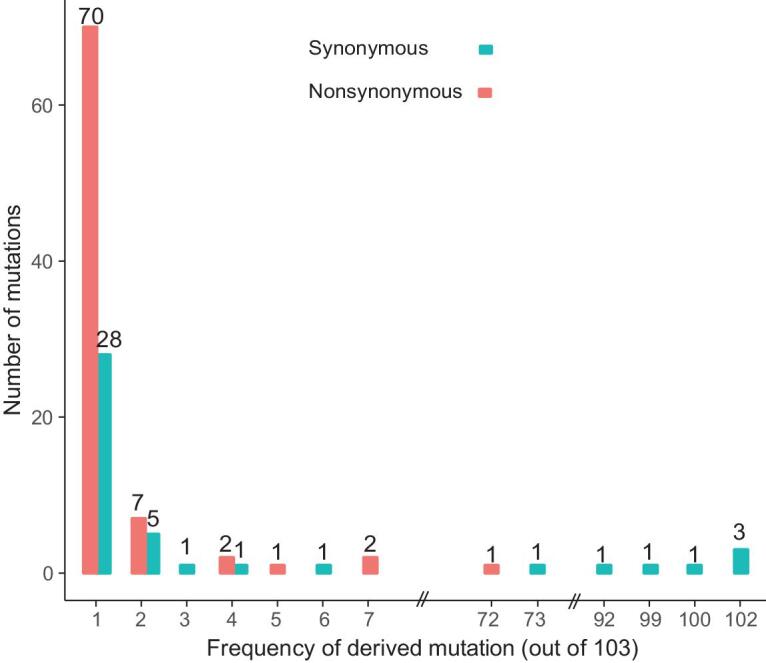
The frequency spectra of derived mutations in 103 SARS-CoV-2 viruses. Note the derived alleles of synonymous mutations are skewed towards higher frequencies than those of nonsynonymous mutations.

We downloaded 103 publicly available SARS-CoV-2 genomes, aligned the sequences, and identified the genetic variants. For ease of visualization, we marked each virus strain based on the location and date the virus was isolated with the format of ‘Location_Date’ throughout this study (see Table S1 for details; Each ID did not contain information of the patient's race or ethnicity). Although SARS-CoV-2 is an RNA virus, for simplicity, we presented our results based on DNA sequencing results throughout this study (*i.e.* the nucleotide T (thymine) means U (uracil) in SARS-CoV-2). For each variant, the ancestral state was inferred based on the genome and CDS alignments of SARS-CoV-2 (NC_045512), RaTG13, and GD Pangolin-CoV (Materials and Methods). In total, we identified mutations in 149 sites across the 103 sequenced strains. Ancestral states for 43 synonymous, 83 non-synonymous, and two stop-gain mutations were unambiguously inferred. The frequency spectra of synonymous and nonsynonymous mutations are shown in Fig. 2.

Most derived mutations were singletons (65.1% (28/43) of synonymous mutations and 84.3% (70/83) of nonsynonymous mutations), indicating either a recent origin [32] or population growth [33]. In general, the derived alleles of synonymous mutations were significantly skewed towards higher frequencies than those of nonsynonymous ones (*P* < 0.01, Wilcoxon rank-sum test; Fig. 2), suggesting the nonsynonymous mutations tended to be selected against. However, 16.3% (7 out of 43) synonymous mutations, and one nonsynonymous (ORF8 (L84S, 28,144)) mutation had a derived frequency of ≥ 70% across the SARS-CoV-2 strains. The nonsynonymous mutations that had derived alleles in at least two SARS-CoV-2 strains affected six proteins: orf1ab (A117T, I1607V, L3606F, I6075T), S (H49Y, V367F), ORF3a (G251V), ORF7a (P34S), ORF8 (V62L, S84L), and N (S194L, S202N, P344S).

### Two major lineages of SARS-CoV-2 defined by two linked SNPs

**Figure 3.**
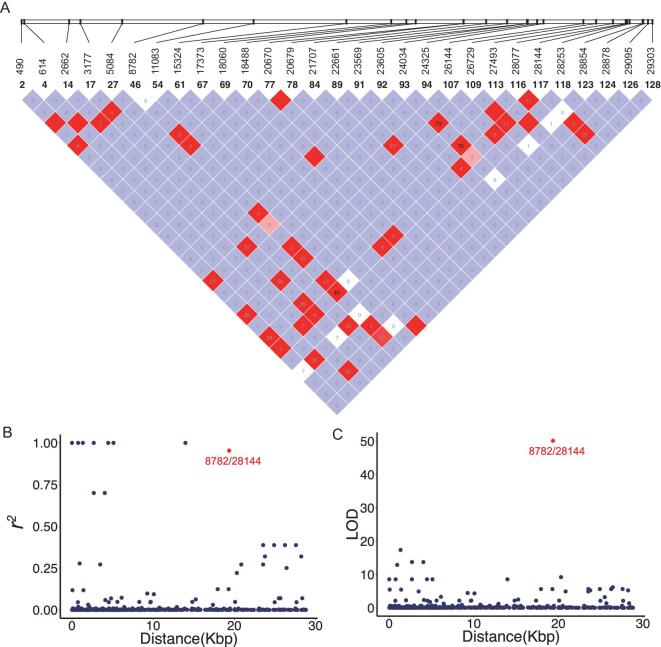
Linkage disequilibrium between SNPs in the SARS-CoV-2 viruses. (A) LD plot of any two SNP pairs among the 29 sites that have minor alleles in at least two strains. The number near slashes at the top of the image shows the coordinate of sites in the genome. Color in the square is given by standard (D'/LOD), and the number in square is *r^2^* value. (B) The *r^2^* of each pair of SNPs (*y*-axis) against the genomic distance between that pair (*x*-axis). (C) The LOD of each pair of SNPs (*y*-axis) against the genomic distance between that pair (*x*-axis). Note that in both (B) and (C), the red point represents the LD between SNPs at 8,782 and 28,144.

To detect the possible recombination among SARS-CoV-2 viruses, we used Haploview [34] to analyze and visualize the patterns of linkage disequilibrium (LD) between variants with minor alleles in at least two SARS-CoV-2 strains (Fig. 3A). Since most mutations were at very low frequencies, it is not surprising that many pairs had a very low *r^2^* or LOD value (Fig. 3B and C). Consistent with a recent report [33], we did not find evidence of recombination between the SARS-CoV-2 strains.

However, we found that SNPs at location 8,782 (*orf1ab*: T8517C, synonymous) and 28,144 (*ORF8*: C251T, S84L) showed significant linkage, with an *r^2^* value of 0.954 (Fig. 3B, red) and a LOD value of 50.13 (Fig. 3C, red). Among the 103 SARS-CoV-2 virus strains, 101 of them exhibited complete linkage between the two SNPs: 72 strains exhibited a ‘CT’ haplotype (defined as ‘L’ lineage because T28,144 is in the codon of Leucine) and 29 strains exhibited a ‘TC’ haplotype (defined as ‘S’ lineage because C28,144 is in the codon of Serine) at these two sites. Thus, we categorized the SARS-CoV-2 viruses into two major lineages with L being the major (∼70%) and S being the minor (∼30%).

### The evolutionary history of L and S lineages

**Figure 4.**
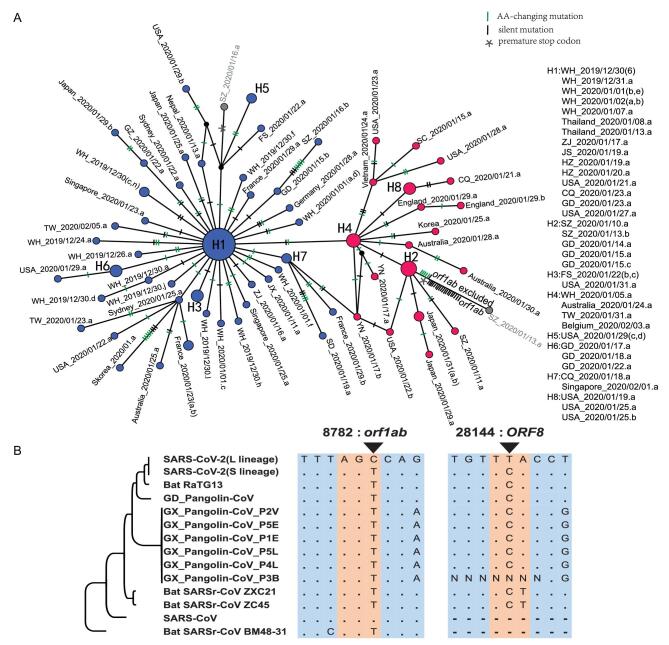
Haplotype analysis of SARS-CoV-2 viruses. (A) The haplotype networks of SARS-CoV-2 viruses. Blue represents the L lineage, and red is the S lineage. Note that in this study, we marked each sample with a unique ID that starting with the geological location, followed by the date the virus was isolated (see Table S1 for details). Each ID did not contain information of the patient's race or ethnicity. ZJ, Zhejiang; YN, Yunnan; WH, Wuhan; USA, United States of America; TW, Taiwan; SZ, Shenzhen; SD, Shandong; SC, Sichuan; JX, Jiangxi; JS, Jiangsu; HZ, Hangzhou; GZ, Guangzhou; GD, Guangdong; FS, Foshan; CQ, Chongqing. (B) Evolution of the L and S lineages of SARS-CoV-2 viruses. ‘.’, The nucleotide sequence is identical; ‘-’, gap.

Although we defined the L and S lineages based on two tightly linked SNPs, strikingly, the separation between the L (blue) and S (red) lineages was maintained when we reconstructed the haplotype networks using all the SNPs in the SARS-CoV-2 genomes (Fig. 4A; the number of mutations between two neighboring haplotypes was inferred parsimoniously). This analysis further supports the idea that the two linked SNPs at sites 8,782 and 28,144 adequately define the L and S lineages of SARS-CoV-2.

To determine the evolutionary changes associated with L and S lineages, we examined the genomic alignment of SARS-CoV-2 and other highly related viruses. Strikingly, nucleotides of the S lineage at sites 8,782 and 28,144 were identical to the orthologous sites in the most closely related viruses (Fig. 4B). Remarkably, both sites were highly conserved in other viruses as well. Hence, although the L lineage (∼70%) was more prevalent than the S lineage (∼30%) in the SARS-CoV-2 viruses we examined, the S lineage was evolutionarily more related to animal coronaviruses.

**Figure 5.**
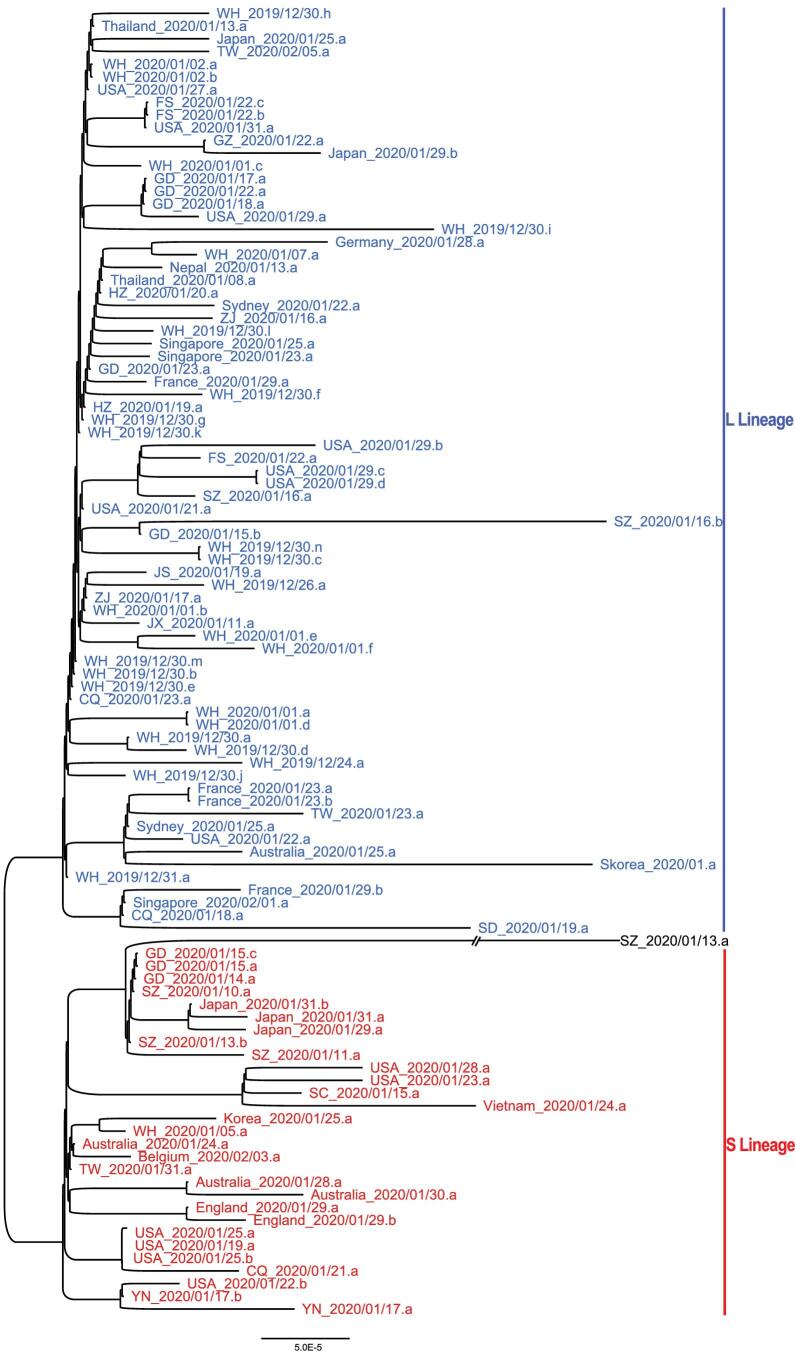
The unrooted phylogenetic tree of the 103 SARS-CoV-2 genomes. The ID of each sample is the same as in Fig. 4A. Note WH_2019/12/31.a represents the reference genome (NC_045512). Note SZ_2020/01/13.a had C at both positions 8,782 and 28,144 in the genome, belonging to neither L nor S lineage.

To further examine the relationship among the strains in the L and S lineages, we reconstructed a phylogenetic tree of all the 103 SARS-CoV-2 viruses based on their whole-genome sequences. Our phylogenetic tree also clearly shows the separation of the two lineages (Fig. 5). Viruses of the L lineage (blue) clustered together, and likewise, viruses of the S lineage (red) were also more closely related to each other. Therefore, our whole-genome comparisons further confirm the separation of the L and S lineages.

Furthermore, our mutational load analysis indicated that the L lineage had accumulated a significantly higher number of derived mutations than S lineage (*P* < 0.0001, Wilcoxon rank-sum test; Fig. S5). Whether the two lineages might have different rates in transmission or replication needs to be investigated in future studies.

These results support notions that two lineages of SARS-CoV-2 viruses may have experienced different selective pressures. Of note, the above analyses were based on limited SARS-CoV-2 genomes that were collected from various locations with different time points. More comprehensive genomic data is required for further testing of our hypothesis.

### Heteroplasmy of SARS-CoV-2 viruses in patients

We found that the sequence of viruses isolated from one patient that lived in the United

**Figure 6.**
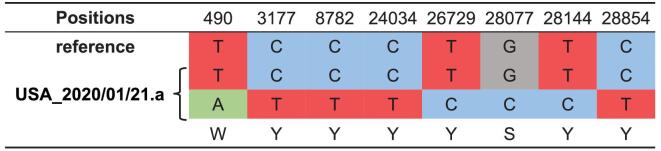
The heteroplasmy of SARS-CoV-2 viruses in human patients. The viruses isolated from a patient that lived in the United States (USA_2020/01/21.a, GISAID ID: EPI_ISL_404253) had the genotype Y (C or T) at both 8,782 and 28,144. The most likely explanation is that this patient was infected by both the L and S lineages. Note the reference is L lineage.

States on January 21 (USA_2020/01/21.a, GISAID ID: EPI_ISL_404253) had the genotype Y (C or T) at both positions 8,782 and 28,144, differing from the general trend of having either C or T. Although novel mutations could lead to this result, the most parsimonious explanation is that this patient may have been infected by both the L and S lineages (Fig. 6). The sample of USA_2020/01/21.a was collected from a 63-year-old female patient living in Chicago (from GISAID). Based on the report from the United States Centers for Disease Control and Prevention (https://www.cdc.gov/media/releases/2020/p0124-second-travel-coronavirus.html).

To further investigate the heteroplasmy of SARS-CoV-2 viruses in patients, we searched 12 deep-sequencing libraries of SARS-CoV-2 genomes that were deposited in the Sequence Read Archive (SRA) (Table S3, Materials and Methods). We found 17 genomic sites that showed evidence of heteroplasmy of SARS-CoV-2 virus in five patients, but we did not find any other instances of the co-existence of L and S lineages in any patient (Table 2). These findings point to the complexity of SARS-CoV-2 evolution. Further studies investigating how the different alleles of SARS-CoV-2 viruses compete with one and another will be of significant value.

**Table 2.** The heteroplasmy of SARS-CoV-2 viruses in human patients.

Accession number	Genomic position	Ref allele	Alt allele	Ref reads	Alt reads	Location_date	GISAID ID
SRR10903401	1821	G	A	52	5	WH_2020/01/02.a	EPI_ISL_406716
SRR10903401	19164	C	T	40	12	WH_2020/01/02.a	EPI_ISL_406716
SRR10903401	24323	A	C	102	67	WH_2020/01/02.a	EPI_ISL_406716
SRR10903401	26314	G	A	15	2	WH_2020/01/02.a	EPI_ISL_406716
SRR10903401	26590	T	C	10	2	WH_2020/01/02.a	EPI_ISL_406716
SRR10903402	11563	C	T	164	26	WH_2020/01/02.b	EPI_ISL_406717
SRR11092057	9064	TTAT	TT	13	2	WH_2019/12/30.e	EPI_ISL_402124
SRR11092057	17825	C	T	19	5	WH_2019/12/30.e	EPI_ISL_402124
SRR11092059	4795	C	T	10	4	WH_2019/12/30.h	EPI_ISL_402130
SRR11092059	6360	A	G	39	5	WH_2019/12/30.h	EPI_ISL_402130
SRR11092059	7042	G	A	5	3	WH_2019/12/30.h	EPI_ISL_402130
SRR11092059	12153	C	T	15	13	WH_2019/12/30.h	EPI_ISL_402130
SRR11092059	15921	G	T	19	2	WH_2019/12/30.h	EPI_ISL_402130
SRR11092059	16474	A	G	11	2	WH_2019/12/30.h	EPI_ISL_402130
SRR11092059	20344	C	T	19	2	WH_2019/12/30.h	EPI_ISL_402130
SRR11092062	565	T	C	64	23	WH_2019/12/30.e	EPI_ISL_402124
SRR11092062	17825	C	T	141	34	WH_2019/12/30.e	EPI_ISL_402124
SRR11092063	29441	C	A	6	2	WH_2019/12/30.d	EPI_ISL_402127

## DISCUSSION

In this study, we investigated the patterns of molecular divergence between SARS-CoV-2 and other related coronaviruses. Although the genomic analyses suggested that SARS-CoV-2 was closest to RaTG13, their difference at neutral sites was much higher than previously realized. Our results provide novel insights for tracing the intermediate natural host of SARS-CoV-2. With population genetic analyses of 103 genomes of SARS-CoV-2, we found that SARS-CoV-2 viruses had two major lineages (L and S lineages), and the two lineages were well defined by just two SNPs that show complete linkage across SARS-CoV-2 strains. The L lineage (∼70%) was found to be more prevalent than the S lineage (∼30%) in the SARS-CoV-2 viruses we examined, our evolutionary analyses suggested the S appeared to be more related to coronaviruses in animals.

Since nonsynonymous sites are usually under stronger negative selection than synonymous sites, calculating sequence differences without separating these two classes of sites could lead to a potentially significant underestimate of the degree of molecular divergence. For example, although the overall nucleotides only differed by ∼4% between SARS-CoV-2 and RaTG13, the genomic average dS value, which is usually a neutral proxy, was 0.17 between these two viruses (Table 1). Of note, the genome-wide dS value is 0.012 between humans and chimpanzees [35], and 0.08 between humans and rhesus macaques [36]. Thus, the neutral molecular divergence between SARS-CoV-2 and RaTG13 is 14 times larger than that between humans and chimpanzees, and twice as large as that between humans and macaques. The genomic average dS value between SARS-CoV-2 and GD Pangolin-CoV is 0.469, which is comparable to that between humans and mice (0.5) [37], and the dS value between SARS-CoV-2 and GX Pangolin-Cov is even larger (0.722). The scale of these measures suggests that we should perhaps consider the difference in the neutral evolving site rather than the difference in all nucleotide sequences when tracing the origin and natural intermediate host of SARS-CoV-2.

In this work, we propose that SARS-CoV-2 can be divided into two major lineages (L and S). Intriguingly, the S and L lineages can be clearly defined by just two tightly linked SNPs at positions 8,782 (*orf1ab*: T8517C, synonymous) and 28,144 (*ORF8*: C251T, S84L). *orf1ab*, which encodes replicase/transcriptase, is required for viral genome replication and might also be important for viral pathogenesis [38]. Although the T8517C mutation in *orf1ab* does not change the protein sequence (it changes the codon AGT (Ser) to AGC (Ser)), it may affect *orf1ab* translation since AGT is preferred while AGC is unpreferred (Table S2). ORF8 promotes the expression of ATF6, the ER unfolded protein response factor, in human cells [39]. Thus, it will be interesting to investigate the function of the S84L AA change in ORF8, as well as the combinatory effect of these two mutations in SARS-CoV-2 pathogenesis.

As previously noted [19], the data examined in this study are still very limited, and follow-up analyses of a larger set of data are needed to have a better understanding of the evolution and epidemiology of SARS-CoV-2.

## MATERIALS AND METHODS

### Molecular evolution of SARS-CoV-2 and other related viruses

The set of 103 complete genome sequences were downloaded from GISAID (Global Initiative on Sharing All Influenza Data; https://www.gisaid.org/) with acknowledgment, GenBank (https://www.ncbi.nlm.nih.gov/genbank), and NMDC (http://nmdc.cn/#/nCoV). Sequences and annotations of the reference genome of SARS-CoV-2 (NC_045512) and other related viruses were downloaded from GenBank, GISAID or Genome Warehouse. The two genomes of coronavirus from Guangdong Pangolins were downloaded from GISAID (EPI_ISL_410544) and Genome Warehouse (GWHABKW00000000; see Table S1 for acknowledgement). We merged them to build the consensus sequence. The genomic sequences of SARS-CoV-2 were aligned using MUSCLE v3.8.31 [40].

The annotated CDSs of other viruses were downloaded from GenBank. To avoid missing annotations in other viruses, we also annotated the ORFs using CDSs annotated in SARS-CoV-2 using Exonerate (–model protein2genome: bestfit –score 5 -g y) [41]. The protein sequences of SARS-CoV-2 and other related viruses were aligned with MUSCLE v3.8.31 [40], and the codon alignments were made based on the protein alignment with RevTrans [42]. The codon alignments of the conserved ORFs were further concatenated for down-stream evolutionary analysis. The phylogenetic tree was constructed by the neighbor-joining method in MEGA-X [43] using the parameters of Kimura 2-parameter model, and only the third positions of codons were considered. YN00 from PAML v4.9a [22] was used to calculate the pairwise divergence between SARS-CoV-2 and other viruses for each individual gene or for the concatenated sequences. The free-ratio model in CODEML in the PAML [22] package was used to calculate the dN, dS, and ω values for each branch.

### Positively selected amino acids

Positive selection was detected using EasyCodeML [44], a recently published wrapper of CODEML [22]. The M7 and M8 models were compared. In the M7 model, ω follows a beta distribution such that 0 ≤ ω ≤ 1, and in the M8 model, a proportion p_0_ of sites have ω drawn from the beta distribution, and the remaining sites with proportion p_1_ are positively selected and have ω_1_ > 1. The LRTs between M7 and M8 models were conducted by comparing twice the difference in log-likelihood values (2 ln Δl) against a χ^2^-distribution (df = 2). The positively selected sites were identified with the Bayes Empirical Bayes (BEB) score larger than 0.95.

### Haplotype network

DnaSP v6.12.03 [45] was used to generate multi-sequence aligned haplotype data, and PopART v1.7 [46] was used to draw haplotype networks based on the haplotypes generated by DnaSP. RAxML v8.2.12 [47] was used to build the maximum likelihood phylogenetic tree of 103 aligned SARS-CoV-2 genomes with theparameters ‘-p 1234 -m GTRCAT’.

### SNP calling process

We downloaded 12 SARS-CoV-2 metagenomic sequencing libraries (Table S2), and mapped the NGS reads to the reference genome of SARS-CoV-2 (NC_045512) using BWA (0.7.17-r1188) [48] with the default parameters. SNP calling was done using bcftools mpileup (bcftools 1.9) [49].

### Codon usage bias analysis

We calculated the RSCU (Relative Synonymous Codon Usage) value of each codon in the SARS-CoV-2 reference genome (NC_045512). The RSCU value for each codon was the observed frequency of this codon divided by its expected frequency under equal usage among the amino acid [50]. The codons with RSCU > 1 were defined as preferred codons, and those with RSCU < 1 were defined as unpreferred codons. The FOP (frequency of optimal codons) value of each gene was calculated as the number of preferred codons divided by the total number of preferred and unpreferred codons.

## Supplementary Material

nwaa036_Supplemental_File
